# Assessment of the immunogenicity and protection of a Nipah virus soluble G vaccine candidate in mice and pigs

**DOI:** 10.3389/fmicb.2022.1031523

**Published:** 2022-10-06

**Authors:** Zihan Gao, Tao Li, Jicheng Han, Sheng Feng, Letian Li, Yuhang Jiang, Zhiqiang Xu, Pengfei Hao, Jing Chen, Jiayi Hao, Peng Xu, Mingyao Tian, Ningyi Jin, Weijin Huang, Chang Li

**Affiliations:** ^1^Research Unit of Key Technologies for Prevention and Control of Virus Zoonoses, Chinese Academy of Medical Sciences, Changchun Institute of Veterinary Medicine, Chinese Academy of Agricultural Sciences, Changchun, China; ^2^Division of HIV/AIDS and Sex-transmitted Virus Vaccines, Institute for Biological Product Control, National Institutes for Food and Drug Control (NIFDC) and WHO Collaborating Center for Standardization and Evaluation of Biologicals, Beijing, China

**Keywords:** adjuvant vaccine, G glcoyprotein, livestock, Nipah vaccine, protein vaccine, zoonosis

## Abstract

Nipah virus (NiV) is a newly emerged extremely dangerous zoonotic pathogen highly fatal to humans. Currently, no approved vaccine is available against NiV. This study employed a mammalian eukaryotic system to express NiV soluble G glycoprotein (NiV-sG), using CpG oligodeoxynucleotides (CpG)/Aluminum salt (Alum) as adjuvants to obtain a recombinant subunit vaccine candidate. We also evaluated the immunogenicity and efficacy of the protein in mice and pigs. The results showed that humoral and cellular immune responses were induced in all the vaccination groups in two animal models. The levels of specific and neutralizing antibodies and the proliferation levels of T helper(Th) cells were significantly higher than those in the control group. The protective efficacy of the subunit vaccines evaluated in the pseudovirus *in vivo* infection mouse model strongly suggested that this vaccine could provide protective immunity against NiV. A neoadjuvant (HTa) based on liposomes and cholera toxin combined with CpG/Alum was exploited and evaluated in mice. The neoadjuvant group showed a more protective efficacy than the CpG/Alum group. The aforementioned results indicated that the subunit vaccine could be used as a promising candidate vaccine for preventing Nipah virus infection.

## Introduction

Nipah virus (NiV), as a newly emerged zoonotic virus, is a single-stranded negative-strand RNA virus belonging to the *Paramyxoviridae* family. It is a member of the genus *Henipavirus*, with Hendra virus (Chua et al., 2000; HeV), Cedar virus (Marsh et al., 2012; CedPV), and Mojiang virus (Li et al., 2020)(MojV) in the same genus. Nipah virus and Hendra virus are classified as biosafety level 4 viruses due to the high fatality rate in humans (40–70%; Lo Presti et al., 2016). The virus has a broad species tropism perhaps. Its main host is a fruit bat (Luby et al., 2009), but a pig is the amplified host in Malaysia. The clinical symptoms in pigs infected with NiV are not uniform (Nor et al., 2000), making the detection of viral infections difficult. This condition increases the probability of virus transmission from pigs to humans and poses a huge threat to pig farmers and related industry workers. The Nipah virus outbreak in Malaysia first broke out in 1998. The government culled nearly half of the country’s live pigs (>1,000,000) as the cost for controlling the epidemic (Parashar et al., 2000); NiV has also been proven to be transmitted from person to person (Kim, 2011). More than half of the cases in Bangladesh are caused by person-to-person transmission (Satterfield et al., 2016). Although NiV is a new pathogen of the disease deserving attention, no therapies or vaccines are approved for use in humans or susceptible animals except for horses. Therefore, the development of vaccines and treatment methods is urgent and necessary.

Considering the rapid onset of the Nipah virus disease, the high probability of death, and the irreversibility of some neurological sequelae caused by acute encephalitis (Sejvar et al., 2007), preventing the spread of the virus is the primary goal of scientific researchers. One of the most important strategies to prevent viral infections is the use of vaccines. In terms of antigen selection for the development of NiV vaccines, most of these antigens are the two main surface glycoproteins of NiV: attachment glycoprotein (NiV-G) and fusion glycoprotein (NiV-F). Previous studies found that NiV-G binds to the ephrin-B2/-B3 receptor, and NiV-F drives membrane fusion for viral entry and the formation of intercellular syncytia (Bonaparte et al., 2005; Negrete et al., 2005). Since then, NiV-G is considered to be a key antigen for the development of NiV vaccines. At present, many vaccines using NiV-G as antigens are under development (McLean and Graham, 2019). Only one recombinant NiV vaccine based on Canarypox vectors (ALVAC) carrying the gene of the NiV-G/F instead of HeV-G/F has been shown to protect pigs from NiV challenges (Weingartl et al., 2006).

For the development of subunit vaccines, the selection and use of adjuvants are critical to whether the vaccine can elicit a protective immune response. CpG ODN and aluminum ion are commonly used adjuvant combinations for subunit vaccines. As an agonist of TLR9 (Class B), CpG could strongly activate TLR9-mediated NF-B signaling, showing Th1 adjuvant effects (Hartmann et al., 2000; Dan et al., 2014), while aluminum adjuvants stimulated robust Th2 responses (Marrack et al., 2009). However, our previous results indicated that a combination of CpG ODN and aluminum ions elicited a Th2-biased immune response. We hoped that Th1/Th2 could be balanced by adding a complex adjuvant (HTa adjuvant). HTa is a complex adjuvant of cholera toxin linked by a liposome carrier. The liposomes are composed of self-assembled lipids and include the delivery of mono-lipopeptides, di-lipopeptides, and their mixtures to elicit and modulate cellular immunity.

In this study, based on the soluble G (sG) glycoprotein expressed by the Expi293F expression system, we used a CpG oligodeoxynucleotide (CpG) containing a fully phosphorothioate backbone and aluminum salt as an adjuvant to evaluate the humoral and cellular immune responses in mice and piglets. The result demonstrated that the CpG/aluminum ion adjuvant vaccine candidate (SAC) induced robust humoral immunity and significant Th cell proliferation in both species. A neoadjuvant (HTa) combined with CpG/Alum (SACF) was used in the mouse model. The result showed higher specific immunoglobulin G (IgG) and neutralizing antibody titers compared with the CpG/Alum adjuvant vaccine at 28 dpi. Moreover, *in vivo* neutralization experiments with pseudoviruses in mice, the result showed a more pronounced difference in comparing the SAC group with the control group. In conclusion, the CpG/aluminum adjuvant vaccine candidates have the potential to enter the preclinical stage, and the good performance of the SACF also provides a new insight for the research and development of this subunit vaccine.

## Materials and methods

### Cells

Expi293F [(Gibco, NY, United States), A14527], 293 T [American Type Culture Collection (ATCC), CRL-3216], and Vero [American Type Culture Collection (ATCC), FS-0393] cells were used in the study.

### Preparation of the vaccine

The expression and purification of NiV soluble G protein (NiV-sG) were performed as described previously (Li et al., 2020), but with some modifications. The sequence of the NiV-G protein came from the representative strain of Nipah (Chua et al., 2000; Genbank: AF212302.2). The Expi293 expression system (Thermo Fisher Scientific, MA, United States) was used to express this protein. We added Kozak, tPA signal peptide, twin-strep-tag, and TEV protease site at the N-terminus of the sG protein sequence (G protein extracellular domain), the size of protein was about 60 kDa (Supplementary Figure S1A). The aforementioned sequences were codon optimized, synthesized, and inserted into the pcDNA3.1 (+) mammalian expression vector (Invitrogen, CA, United States), and the protein was purified using the Strep-Tactin Superflow high-capacity column (IBA Lifesciences GmbH, Göttingen, Germany; Supplementary Figure S1B). The mouse-specific anti-sG protein serum previously prepared by our laboratory was used as the primary antibody to characterize the antigen by Western blot (WB) analysis. The nonreduced SDS-polyacrylamide gel electrophoresis (SDS-PAGE) and thin-layer chromatography scanner were used to determine the purity of the antigen (Supplementary Figure S1C). CpG oligodeoxynucleotide (ODN) containing a fully phosphorothioate backbone and aluminum ion (Alum; Thermo Fisher Scientific) as an adjuvant. The sG was mixed with Alum and shaken on a vortex shaker for 5 min. Then, CpG ODN was added, and finally PBS was used to make up to the required final concentration.

### Animal immunization

**Figure 1:**
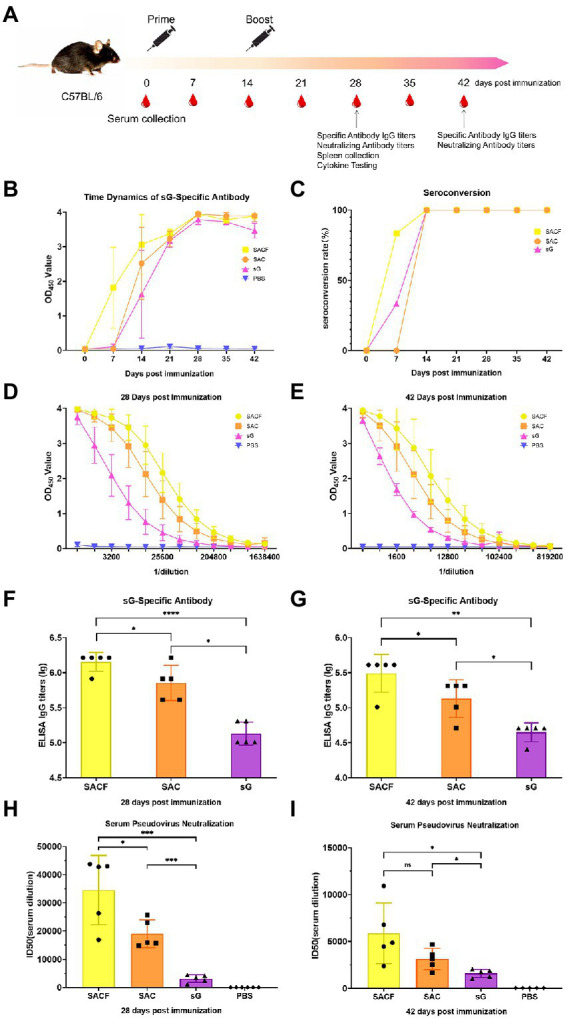
Vaccine candidate induced humoral response in the C57/BL6 mouse model. **(A)** Immunization procedure and vaccine candidate efficacy evaluation in mice. **(B)** OD value of NiV-sG-specific IgG antibody over time determined using ELISA. Error bars represent standard deviation from the mean of five biological replicate values. **(C)** Seroconversion over time calculated from the date of B. **(D,E)** OD values of serial dilution of serum at 28 and 42 dpi using ELISA. Error bars represent standard deviation from the mean of five biological replicate values. **(F,G)** NiV-sG specific IgG antibody titers of serum at 28 and 42 dpi; five dots represent five biological replicates. Error bars represent standard deviation from the mean of five replicate values (*p < 0.05, **p < 0.01, ***p < 0.001, ****p < 0.0001). **(H,I)** Neutralizing antibody titers determined using serum (collected from mice at 28 and 42 dpi) pseudovirus neutralization assay. Data indicated by log10, which were from five biological replicates; each dot performed in triplicate. Error bars represent standard deviation from the mean of five replicate values (*p < 0.05, **p < 0.01, ***p < 0.001, ****p < 0.0001). The Student *t* test was used in the analysis of significant differences between the groups.

Mice: 7-week-old female C57BL/6 mice were randomly divided into four groups (*n* = 10). We set up four groups: SACF, SAC, sG, and PBS (Table 1). The injection method was intramuscular. A Prime-boost immunization strategy was employed, with boost immunization 2 weeks after the first immunization. The serum was collected at 0, 7, 14, 21, 28, 35, and 42 dpi. Five randomly selected mice were sacrificed at 28 dpi, and spleen lymphocytes were separated (Figure 1A).

**Table 1:** Immune groups.

Group	Treatment	Animals	Number	Antigen	Adjuvant	Immunization times
SACF	H	C57BL/6	5	sG(10 μg)	CpG/Alum/Hta(10 μg/100 μg/200 μl)	2
SAC	H	C57BL/6	5	sG(10 μg)	CpG/Alum(10 μg/100 μg/200 μl)	2
sG	H	C57BL/6	5	sG(10 μg)	—	2
PBS	H	C57BL/6	5	PBS	—	2
SACF	C	C57BL/6	5	sG(10 μg)	CpG/Alum/Hta(10 μg/100 μg/200 μl)	2
SAC	C	C57BL/6	5	sG(10 μg)	CpG/Alum(10 μg/100 μg/200 μl)	2
sG	C	C57BL/6	5	sG(10 μg)	—	2
PBS	C	C57BL/6	5	PBS	—	2
SACF	I	BALB/c	4	sG(10 μg)	CpG/Alum/Hta(10 μg/100 μg/200 μl)	2
SAC	I	BALB/c	4	sG(10 μg)	CpG/Alum(10 μg/100 μg/200 μl)	2
sG	I	BALB/c	4	sG(10 μg)	—	2
PBS	I	BALB/c	4	PBS	—	2
SAC-HD	H/C	Landrace × York	3	sG(250 μg)	CpG/Alum(250 μg/2500 μg)	2
SAC-LD	H/C	Landrace × York	3	sG(500 μg)	CpG/Alum(250 μg/2500 μg)	2
PBS	H/C	Landrace × York	3	PBS	—	2

Study schedule for the vaccine trials. Indicated are the evaluation treatment for different groups, H, Humoral immune response evaluation; C, Cellular immune response evaluation; I, Injection of pseudovirus.

**Figure 2:**
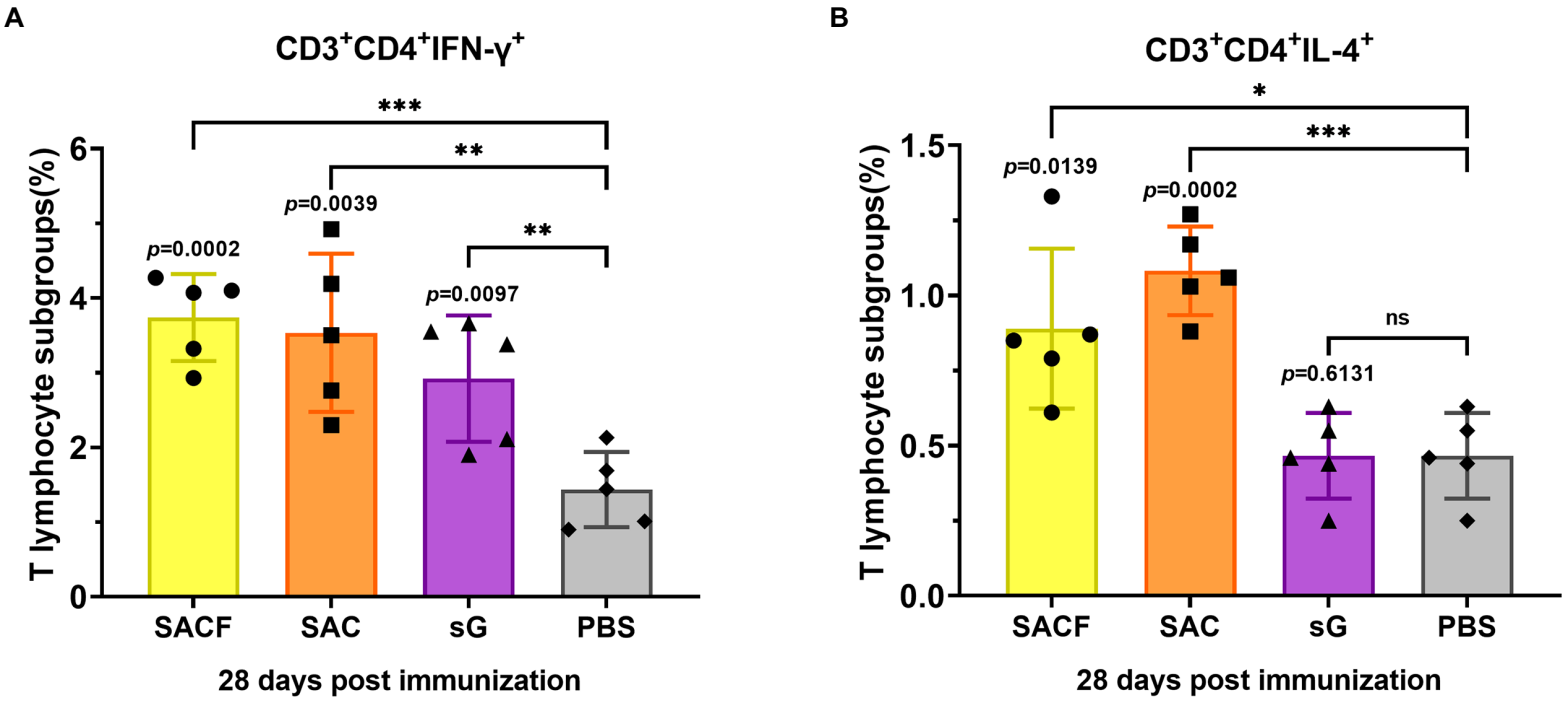
Protective effect of active immunization. **(A)** Process of immune protection in mice. The mice were divided into four groups: SACF, SAC, SG, and PBS control groups. Two immunizations were performed 2 weeks apart. NiV pseudovirus attack was performed 28 days after the initial immunization, and the pseudovirus dose for the *in vivo* infection test was determined as 50 AID50 (the 50% animal infectious dose), equivalent to a dose of 4.4 × 106 TCID50. The luminescent signal was detected 5 days after infection. **(B)** Detection of luminescence signal in each male mouse. Mice immunized with PBS were used as controls. The intensity represented the degree of NiV pseudovirus infection. **(C)** Average radiance flux data were statistically generated in the IVIS Lumina III imaging system, representing the mean intensity of the pseudovirus infection signal. The flux signals in each group were compared with those in the PBS control group using the Student *t* test (*p < 0.05, **p < 0.01, ***p < 0.001).

7-week-old female BALB/c mice were randomly divided into four groups (*n* = 4) and used for *in vivo* imaging after the pseudovirus injection. The grouping and immunization schedules were the same as for C57BL/6 (Table 1). The mice were injected with pseudovirus at 28 dpi. The *in vivo* imaging was performed 2 days later (Figure 2A).

**Figure 3:**
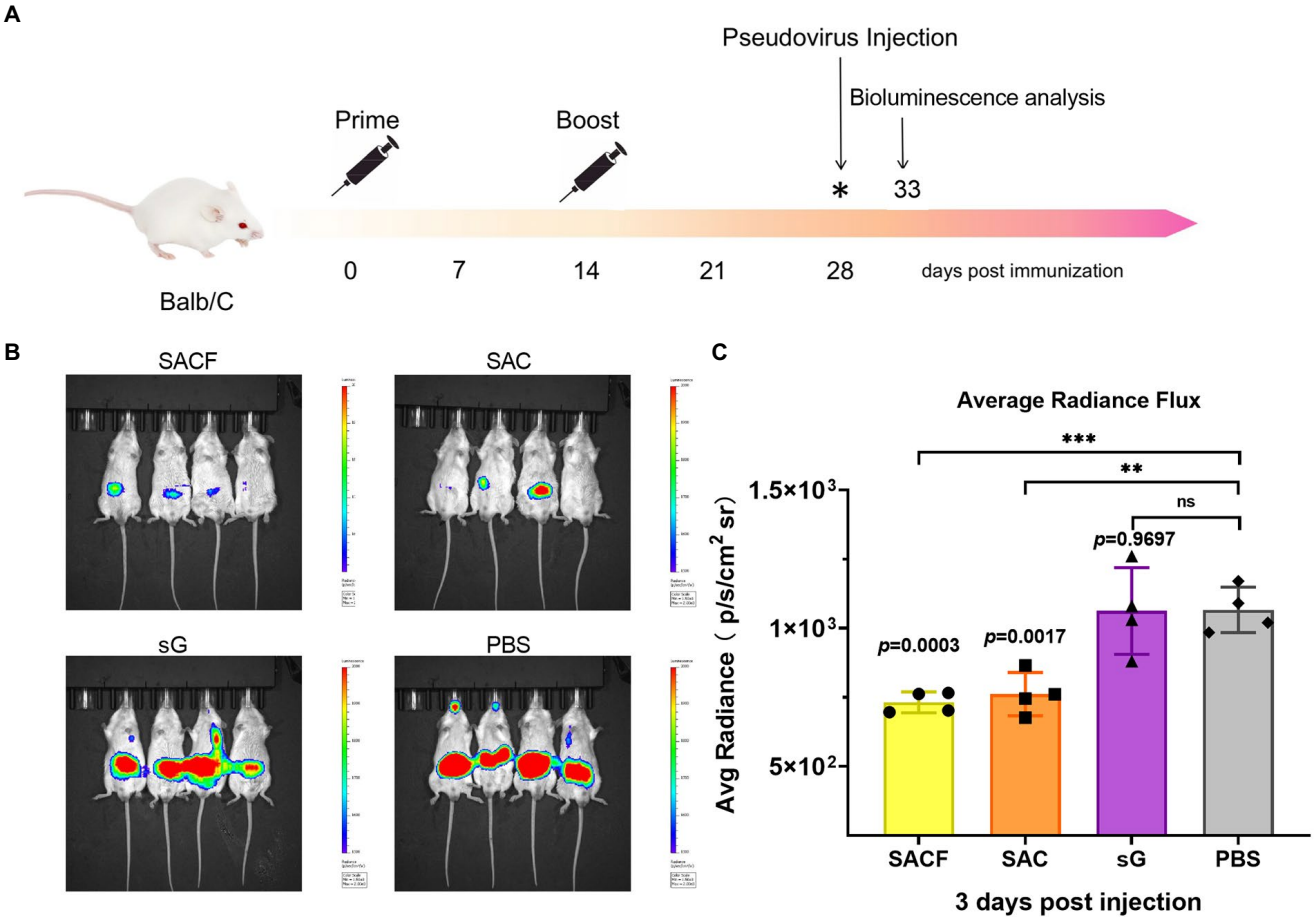
Vaccine candidate induced humoral response in the Landrace × York pig model. **(A)** Immunization procedure and vaccine candidate efficacy evaluation in pigs. **(B)** OD value of NiV-sG-specific IgG antibody over time determined using ELISA. Error bars represent standard deviation from the mean of three biological replicate values. **(C)** Seroconversion over time calculated from the date of B. **(D,E)** OD values of the serial dilution of serum at 35 and 49 dpi using ELISA. Error bars represent standard deviation from the mean of three biological replicate values. **(F,G)** NiV-sG-specific IgG antibody titers of serum at 35 and 49 dpi; three dots represent three biological replicates. Error bars represent standard deviation from the mean of three replicate values (ns *p* >0.05). **(H,I)** Neutralizing antibody titers determined using serum (collected from pigs at 35 and 49 dpi) pseudovirus neutralization assay; the data indicated by log10, which were from three biological replicates; each dot performed in triplicate. Error bars represent standard deviation from the mean of three replicate values (ns *p* >0.05).

Pig: Crossbreed F1 (Landrace × York) female piglets (weaned at 3 weeks of age) were randomly divided into three groups (*n* = 3). We set up experimental groups with high and low doses according to the antigen (NiV-sG) content of each immunization (SAC-HD, SAC-LD), and a dilution control group (PBS; Table 1). The process was roughly the same as in mice, except boost immunization 3 weeks after the first immunization. The serum was collected at 0, 7, 14, 21, 28, 35, 42, and 49 dpi. Three whole-blood samples were randomly selected at 35 dpi to separate peripheral blood lymphocytes (Figure 3A).

### Elisa

The mouse sera were taken at 0, 7, 14, 21, 28, 35, and 42 dpi. The mouse NiV-sG specific antibody IgG kit instructions (Darui Technology, China) were followed to determine the seroconversion rate (the OD values of the experiment group sera were > 2.1-fold of that of the PBS control sera, which indicated seroconversion). Serial dilutions were performed 2 or 4 weeks after terminal immunization (28 and 42 dpi) to determine the specific antibody titer. The titers were determined using the maximum dilution when the OD value in the experimental group was greater than 2.1 times that in the PBS group. The mouse experiment was repeated five biological times for each group. The piglet experiments were consistent with the previous experiments. All sera were collected and tested in accordance with the kit instructions. The specific antibody titer was determined 2 or 4 weeks after terminal immunization (35 and 49 dpi). Pig experiments was performed on 3 biological replicates for each group.

### Production and titration of pseudoviruses

The NiV pseudovirus generation and titration were performed as described previously (Nie et al., 2019). Briefly, HEK-293 T cells were co-transfected with NiV-G and NiV-F envelope protein expression plasmids and HIV packaging vector using Lipofectamine 2000 (Invitrogen, 1,166,819) transfection reagent following the manufacturer’s protocols. After 6-h transfection, the supernatants were discarded and fresh medium was added. The culture supernatants containing NiV pseudovirus were collected 48 h after transfection, filtered (0.45 μm size, Millipore, SLHP033RB), and stored at −80°C. The 50% tissue culture infectious dose (TCID50) of each batch of NiV pseudovirus was determined using aliquots of the samples. All lyophilized samples were used only once to avoid inconsistent results that might result from repeated freeze–thaw cycles. To titrate NiV pseudovirus, fivefold dilutions were performed in sixfold wells of 96-well culture plates for a total of 9 dilutions. The last column was used as a cellular control, and no pseudovirus was added. Then, the 96-well culture plates were inoculated with 293 T cells and adjusted to a predetermined concentration. After 48 h at 37°C in a 5% CO_2_ environment, the supernatant culture was discarded, following which 100 μl of luciferase substrate (Promega, WI, United States) was added to each well. After incubation for 2 min at room temperature, luminescence was detected using a Glomax microplate luminometer (Promega). The TCID50 was calculated using the Reed–Muench method as described previously.

### *In vitro* pseudovirus neutralization assay

NiV pseudovirus neutralization was measured by a reduction in luciferase expression, as previously described in the new coronavirus pseudovirus neutralization assay (Li et al., 2022). The serum dilution corresponding to a 50% inhibitory dilution was defined as the amount of half inhibition (ID50). Specifically, first, the relative light units (RLUs) of all wells were subtracted from the RLUs of the control wells containing only cells. Then, the serum dilution at which the RLU of the sample wells reached 50% of the value of the virus control wells (virus + cells) was considered the ID50 value. Other variations were recounted. Pseudoviruses were incubated with serially diluted serum samples (diluted in a threefold stepwise manner) for 1 h at 37°C, with both virus control and cell control wells set up. Then, 5 × 10^4^ 293 T cells were added to each 96-well plate. After 48 h of incubation at 37°C in the presence of 5% CO_2_, luminescence was measured as described in the pseudovirus titration method. The amount of half inhibition (ID50) was calculated using the Reed–Muench method. Mouse experiments were repeated 15 times (five biological and three technical repeats). For pig experiments, nine times replicates were performed. (three biological and three technical repeats).

### *In vivo* bioluminescence imaging analysis

Bioluminescence analysis was performed using an IVIS Lumina III imaging system (Xenogen, MD, United States) as previously described (Nie et al., 2017). Briefly, the mice were anesthetized by intraperitoneal (IP) injection of sodium pentobarbital (40 mg/kg body weight) followed by IP injection of D-luciferin (150 μg/g body weight; Xenogen Caliper, CA, United States). The bioluminescence signal was detected for each mouse with a 1-min acquisition time 10 min later. The relative bioluminescence was calculated using the photon per second mode as described previously and normalized to the imaging area (photons/s/cm^2^/sr) setting. The experiment was repeated four biological times.

### Analysis of mouse Th1 and Th2 proliferation

At 28 dpi, mouse spleen lymphocytes (*n* = 5) were separated following the instructions on the mouse spleen lymphocyte separation kit (Haoyang Biological Manufacturers, Tianjin, China). Briefly, after counting the obtained lymphocytes, they were diluted with RPMI 1640 medium (Gibco, NY, United States) to 1 × 10^6^ cells/mL. Then, 2 × 10^6^ cells were added per 2.0-mL Eppendorf tube, centrifuged at 2500 rpm, and washed with cell staining buffer (BioLegend, CA, United States). After centrifuging and discarding the supernatant, the diluted CD3 CD4 fluorescent antibody (Invitrogen) was added to each tube (following the manufacturer’s recommended dosage) incubated for 45 min at 4°Cin the dark room and then washed twice. The supernatant was discarded. The fixation/permeabilization buffer (BD Biosciences, CA, United States) was added for 30 min at 4°C. The cells were washed twice with 1× prewash buffer (BD Biosciences) and divided into two tubes. Then, a diluted INF-γ fluorescent antibody was added to one tube and IL-4 fluorescent antibody was added to the other tube. The cells were protected from light for 45 min at 4°C and then washed once with 1× prewash buffer. The supernatant was discarded and the staining buffer was added to resuspend the cells. The analysis was performed using flow cytometry (Bachem, Bubendorf, Switzerland). The mouse experiment was repeated five biological times for each group. Further statistical analysis was used by FlowJo v10 and GraphPad Prism 8.

### Analysis of pig CD3 + CD4+ and CD3 + CD8+ T lymphocytes

At 35 dpi, pig lymphocytes (*n* = 3) were separated following the instructions on the pig peripheral blood lymphocyte separation kit (Hao Yang Biological Manufacturers, Tianjin, China). Most manipulations were consistent with mouse experiments, but no staining for intracellular cytokines was performed. We switched to a strategy whereby the detection of intracellular factors was performed using ELISA, and the CD3^+^CD4^+^/CD3^+^CD8^+^T cells were analyzed using flow cytometry (Bachem, Bubendorf, Switzerland). The experiment was repeated three biological times for each group. Further, statistical analysis was performed using FlowJo v10 and GraphPad Prism 8.

## Results

### Humoral immune response induced by the vaccine candidate in mice

Enzyme-linked immunosorbent assay (ELISA) and serum pseudovirus neutralization assay were used to evaluate the humoral immune response of the vaccine (Figure 1A). First, we measured the time dynamics of NiV-sG-specific IgG in mouse serum using ELISA (Figure 1B). The seroconversion rate in the SACF (sG/Hta/CpG/Alum) group was higher at 7 dpi (Figure 1C). The optical density (OD) value was the highest at 28 dpi in all experimental groups: SACF, SAC (sG/CpG/Alum), and sG (sG). Therefore, we used ELISA with serially diluted mouse serum of 28 dpi and 42 dpi to determine the NiV-sG-specific IgG antibody titers. At 28 dpi, the Geometric Mean Titers (GMTs) in the SACF, SAC, and sG groups reached 1,474,560, 819,200, and 143,360, respectively. The titer in the SACF group was significantly higher than those in the SAC and sG groups (*p* = 0.0460), while the titer in the SAC group was significantly higher than that in the sG group (*p* = 0.0172; Figures 1D,F). At 42 dpi, the overall trends were similar to the trend at 28 dpi; their GMT also reached 348,160, 153,600, and 46,080, respectively. The titer in the SACF group was significantly higher than that in the SAC group (*p* = 0.0231) and the sG group (*p* = 0.0012), while the titer in the SAC group was significantly higher than that in the sG group (*p* = 0.0112; Figures 1E,G). Then, we selected 28-dpi and 42-dpi sera to perform a neutralization test with pseudovirus to determine the neutralizing antibody (nAb) titer (indicated using 50% inhibition dose, ID50). The neutralizing antibody GMTs was consistent with the specific antibody titer trend results.

The neutralizing antibody titer at 28 dpi in the 3 groups was 34,543, 19,006, and 3,163, respectively. As expected, no neutralizing antibodies were detected in the phosphate-buffered saline (PBS) control group. The titers in the SACF group was significantly higher than that in the SAC group (*p* = 0.0305) and sG group (*p* = 0.0005), while the titers in the SAC group were significantly higher than that in the sG group (*p* = 0.0001; Figure 1H).

The neutralizing antibody titer in the 3 groups was 5,883, 3,139, and 1,612, respectively, at 42 dpi. The titer in the SACF group was significantly higher than that in the sG group (*p* = 0.0189), while the titer in the SAC group was significantly higher than that in the sG group (*p* = 0.0220). However, the difference between the SACF and SAC groups was no longer significant (*p* = 0.1103; Figure 1I).

### Cellular immune response induced by the candidate vaccine in mice

**Figure 4:**
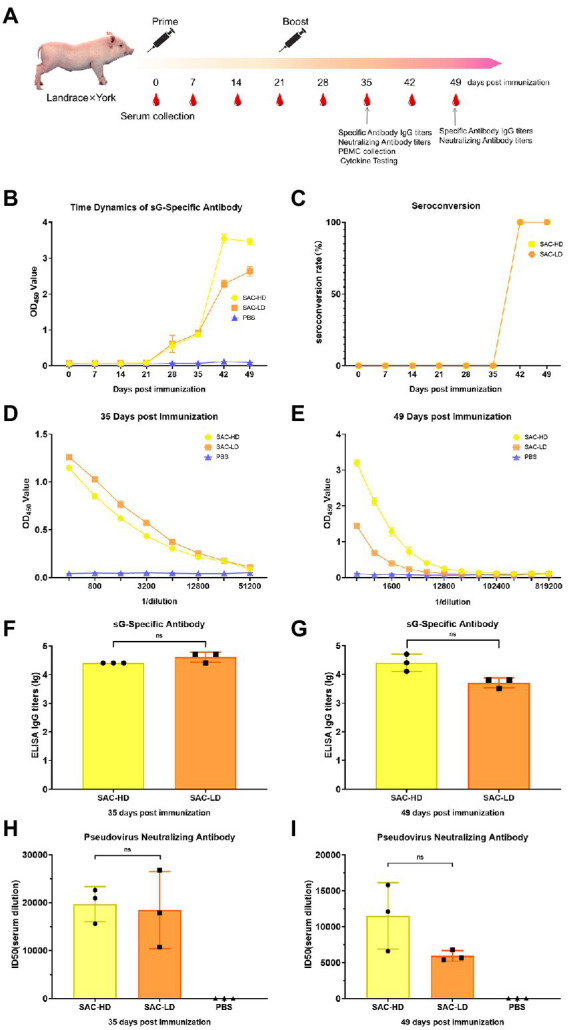
Activation of NiV-sG-specific Th1/2 cells after immunization with candidate vaccine at 28 dpi. **(A)** Data shows the frequency of CD4+ IL-4+ T lymphocytes in CD3 + CD4 + T lymphocytes. **(B)** Data show the frequency of CD4 + IFN-γ + T lymphocytes in CD3 + CD4 + T lymphocytes. The spleen lymphocytes were obtained after the sacrifice of C57BL/6 mice. Five dots represent five biological replicates. Error bars represent standard deviation from the mean of five replicate values (*p < 0.05, **p < 0.01, ***p < 0.001, ****p < 0.0001). The Student *t* test was used to analyze significant differences between the groups.

The cellular immune responses were evaluated by the levels of indicated cell markers and cytokines. For Nipah virus, proliferation of Th cells appears to play a key role in virus clearance (Pickering et al., 2016), and vaccine that induce balanced proliferation of Th1/2 have elicited the best protection in previous reports (Weingartl et al., 2006). Usually, Th1 cells secrete IFN-γ, while Th2 cells secrete IL4. The data showed that the frequency of Th1 cells was significantly higher in the SACF group than in the PBS control group (*p* = 0.0002), while it was significantly higher in the SAC group than in the PBS group (*p* = 0.0039); the difference was more pronounced in SACF. Significantly higher Th1 frequencies were observed in the sG group than in the PBS group (*p* = 0.0097; Figure 4A). Similarly, the frequency of Th2 cells was significantly higher in the SACF group than in the PBS control group (*p* = 0.0139), while it was significantly higher in the SAC group than in the PBS group; the difference was more significant (*p* = 0.0002). The sG and PBS groups showed no significant difference (*p* = 0.6131; Figure 4B).

### Protective efficacy of the candidate vaccines evaluated in the pseudovirus *In vivo* infection model

We evaluate the protective efficacy of the candidate vaccines by measuring the luminescence signals in mice challenged with pseudoviruses. We divided the vaccine candidates into four groups, SACF, SAC, SG, and PBS groups, and then injected the mice in two rounds. Attack with NiV pseudovirus was performed 14 days after completing the booster injections. The intensity represented the degree of NiV pseudovirus infection (Figure 2A). In the *in vivo* protection study, the PBS control group had a significant fluorescence response after pseudovirus attack because it did not induce NiV antibodies, while the other three vaccine candidates reflected different degrees of immune protection. Among these, the mean radiance intensity (representing the mean value of the pseudovirus infection signal) was significantly lower in the SACF and SAC groups than in the PBS control group 5 days after pseudovirus injection (33 dpi; Figure 2B). The difference was more significant (*p* = 0.0003) in the SACF group than in the SAC group (*p* = 0.0017). The pseudovirus infection signal in the sG group was not significantly different from that in the PBS control group (ns, *p* = 0.9697; Figure 2C). The results suggested that the SACF vaccine provided better immune protection.

### Humoral immune response induced by the candidate vaccine in pigs

Two dose groups (SAC-HD and SAC-LD groups) were set up to evaluate the ability of vaccine candidates in pigs (Figure 3A). The same evaluation strategy as in mice was adopted. The time changes in the specific antibody OD value were recorded using ELISA; the OD value in the two groups was the highest at 42 dpi (Figure 3B). The seroconversion rate in both SAC-HD and SAC-LD groups reached 100% at 42 dpi (Figure 3C). The specific IgG titer of GMTs was 25,600 and 42,667 at 35 dpi, respectively, but no significant difference was found between the 2 groups (*p* = 0.1161; Figures 4D,F). At 49 dpi, the specific antibody IgG titer of GMTs in the 2 groups was 29,867 and 5,333, respectively, but still no significant difference was found between the 2 groups (*p* = 0.0965; Figures 3E,G). Then, we performed a pseudovirus neutralizing assay to determine the neutralizing antibody titers at 35 dpi and 49 dpi. The GMTs of neutralizing antibody titers at 35 dpi reached 19,714 and 18,468, respectively, but the SAC-HD and SAC-LD groups did not show significant differences (*p* = 0.8193; Figure 3H). At 49 dpi, the titers of neutralizing antibodies of SAC-HD and SAC-LD groups were 11,514 and 5,930 (*p* = 0.1087), respectively. As expected, no neutralizing antibodies were detected in the PBS group (Figure 3I).

### Cellular immune response induced by the candidate vaccine in pigs

**Figure 5:**
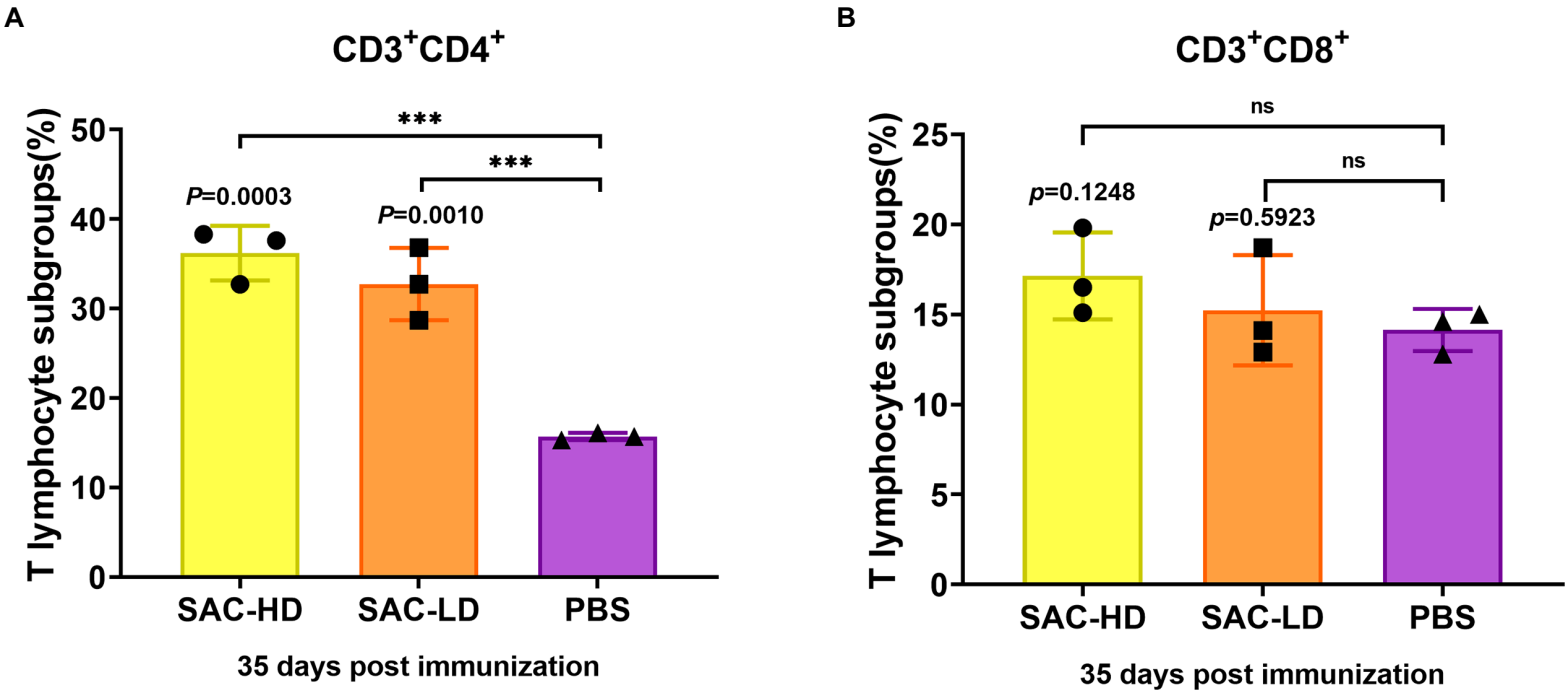
Activation of NiV-sG-specific Th/Tc cells after immunization with candidate vaccine at 28 dpi. **(A)** Data shows the frequency of CD3 + CD4 + T lymphocytes. **(B)** Data show the frequency of CD4 + CD8 + T lymphocytes. The lymphocytes were obtained from the PBMCs of pigs; five dots represent five biological replicates, Error bars represent standard deviation from the mean of five replicate values (*p < 0.05, **p < 0.01, ***p < 0.001, ****p < 0.0001). The Student *t* test was used to analyze significant differences between the groups.

We analyzed the frequency of CD3^+^CD4^+^T (Th, T helper cells) cells and the frequency of CD3^+^CD8^+^T (Tc, cytotoxic T cell) cells using surface marker staining of pig PBMCs at 35 dpi and flow cytometry to investigate whether the candidate vaccine could elicit T cell responses. We found that the frequency of Th cells in the SAC-HD (*p* = 0.0003) and SAC-LD groups (*p* = 0.0010) were significantly higher than that in the PBS control group (Figure 5A), while the Tc cell frequency was not significantly different between the experimental groups and the PBS control group (*p* = 0.1248; *p* = 0.5923; Figure 5B).

### Statistical analysis

Date analysis was used by FlowJo v10 software and GraphPad Prism 8 software (GraphPad. Software Inc.). The Student t-test was used in the analysis of significant differences between the groups, *p* values <0.05 were considered statistically significant (*p < 0.05, **p < 0.01, ***p < 0.001, ****p < 0.0001).

## Discussion

The purpose of this study was to develop a effective vaccine candidate against the Nipah virus, and to evaluate the effects of different adjuvant combinations on immune responses in a mouse and pig model. Rational antigen selection is critical to vaccine development. G glycoprotein is considered an important antigen for NiV vaccine development. Many vaccines that use Nipah virus G protein or G protein + F protein as antigens are under development (Guillaume et al., 2004; Prescott et al., 2015; Singh et al., 2019; Isaacs et al., 2021). The results showed that most neutralizing antibodies were induced by G protein (Weingartl et al., 2006). At present, the only licensed Hendra virus vaccine (Equivac HeV) is based on the protein subunit platform. This showed that the proposed technology was an effective and mature platform for developing Hendra or Nipah virus vaccines.

In this study, a subunit vaccine candidate was constructed based on the expression of NiV-sG protein in a mammalian system (Expi293F), and its immunogenicity and protective efficacy were evaluated in mice and pigs; especially the role of different adjuvants in immune induction was compared. The mouse humoral immune response data showed that high titers of NiV-sG-specific antibodies and neutralizing antibodies were detected in the serum of mice in the SACF (sG/Hta/CpG/Alum) and SAC groups (sG/CpG/Alum). Surprisingly, the specific antibody and neutralizing antibody titers were significantly higher in the SACF group than in the SAC group at 28 dpi: the specific antibody titers in the SACF group could reach 1,474,560, while the neutralizing antibody titers reached 34,543 (Figures 1F,H). In terms of mouse cellular immune response, significant Th1/2 proliferation was detected in the SAC and SACF groups compared with the PBS group. The SACF group had more Th1-biased immune responses, while the SAC group tended toward Th2-type proliferation.

Virus challenge is the most direct and effective method to evaluate vaccines candidates. However, many laboratories do not have pathogens or operating conditions because some highly pathogenic pathogens, such as NiV\HeV, need to be used in BSL-4 laboratories. Hence, various evaluation methods using pseudovirus infection animal models to replace live viruses have been developed (Chen et al., 2018; Tseng et al., 2021; Zhang et al., 2021). Wang and his colleagues (Nie et al., 2019) also established a BALB/c mouse model of NiV pseudovirus infection to evaluate vaccines and antibodies. The present study used this model to further evaluate whether the vaccine induced protective immunity. The results showed that the mean pseudovirus fluorescence intensities in the SACF and SAC groups were significantly different compared with that in the PBS group. The difference was more marked in the SACF group (*p* = 0.0003), indicating that both vaccine candidates provided adequate protection, while the SACF group showed better efficacy. However, the high fluorescence intensity in the sG group indicated that the pure antigen in this evaluation system could not protect the mice, and the specific antibody IgG titer and neutralizing antibody titer also reached 143,360 and 3,163 at 28 dpi, respectively. Nevertheless, in the evaluation of cellular immunity, Th1 cells in the sG group showed significant proliferation, but no significant difference was found in Th2 cells, indicating that moderate but balanced proliferation of Th1 and Th2 cells might be important in vaccine-induced protective responses. In other paramyxoviruses, Th1/2 imbalance caused by viral infection or vaccination could even induce disease deterioration (Lucey et al., 1996; Krishnan et al., 2004), and results demonstrated that the addition of HTa elicited a favorable Th1-biased immune response. The Th2 proliferation induced in the SAC group was more significant, but the serum neutralizing antibody titer and the effect of pseudovirus neutralization assay *in vivo* were not as good as those in the SACF group. It proved that the Th1-biased immune response might mediate better protective immunity, and that HTa might be a potential adjuvant.

Finally, we evaluated the immunogenicity of the vaccine candidates (sG/CpG/Alum) in pigs. The results showed that, both the high- and low-dose groups had high titers of specific IgG antibodies and neutralizing antibodies, similar to that in mice; the neutralizing antibody titers reached 19,714 and 18,468 at 35 dpi, respectively. No significant difference was found between the 2 groups, indicating that the low dose (250 μg) was sufficient to induce a robust humoral immune response. For cellular response evaluation, Th proliferation was detected in the two vaccination groups. Th are important cells that mediate B cell proliferation and differentiation (Zabel et al., 2017; Walker and Mckenzie, 2018), explaining the high titers of antibodies (Kumar et al., 2015). In the only successful vaccine study of the NiV challenge test in pigs (Weingartl et al., 2006), the author used the ALVAC vector to construct three vaccine candidates expressing G, F, and F/G. He found that all pigs in three vaccination groups were protected against the NiV challenge, but the F/G group with Th1/2 activation had the highest neutralizing antibody titer. Another study challenged HeV-sG-vaccinated pigs and NiV-oral-infected pigs with live NiV (Pickering et al., 2016), in the group protected against the NiV challenge (oral infection with live NiV instead of injected vaccine), the upregulation of Th1 marker secretion IFN-γ and Th2 marker secretion IL-10 was detected before the challenge, and the CD4^+^ cell population remained stable in number after the challenge. In piglets that succumbed to experimental infections, a significantly declined frequency of CD4^+^CD8^−^T cells was observed. Both experiments using live NiV-infected pigs showed that the proliferation of Th cells was essential for the formation of a protective immune response in pigs. Limited by the conditions, we could not type Th1/2 as in the pig experiments. The challenge test with live NiV was also not performed, but the subunit vaccine developed in this study did elicit a strong Th-type cell response, showing its protective potential. Two other studies used pseudoviruses to evaluate the neutralizing antibodies of live viral vector vaccine candidates expressing NiV-G or/and F proteins in pigs (Kong et al., 2012; Shuai et al., 2019). The NiV pseudovirus used was constructed with the VSV system. However, the results showed that the neutralizing antibody titers caused by the candidate vaccines in the two studies were not as high as those for the subunit vaccine prepared in this study. The reason was unknown; it might be attributed to the differences in the pseudovirus construction system, but it also indicated that our vaccine might have better protective efficacy. In addition, unlike inactivated, live attenuated vaccines and viral vector vaccines, the subunit vaccine prepared in this study did not involve the cultivation of any live virus. Therefore, the scale-up production and quality control of the protein were simpler, and protein subunit vaccines were easier to store and transport compared with live vector vaccines.

Currently, no approved drugs or vaccines are available for the Nipah virus. Multiple studies reported that the HeV-sG protein could be used in several animals providing protection against NiV [cat (M MCea Chern et al., 2008), ferret (Pallister et al., 2013), and AGM (Bossart et al., 2012; Geisbert et al., 2021)], but successful reports in pigs are lacking: as one of the most important intermediate hosts in Malaysia and Singapore (McLean and Graham, 2022; an important source of meat and the main intermediate host for animal-to-human transmission). Soluble G protein is considered a promising antigen for use against Hendra and Nipah Henipavirus. Further, no relevant experiments have been conducted on the NiV-sG subunit vaccine in pigs (McLean and Graham, 2019). The development and pursuit of a broad-spectrum antiviral vaccine is certainly a promising path, but in special cases for specific viruses and hosts, such as pigs, it is obvious that the HeV-sG does not seem to be so effective. Therefore, developing a vaccine against NiV for pigs is of significance. The main purpose of this vaccine is to prevent not only animal diseases, but more importantly, dangerous zoonotic viruses from shedding from animals and being transmitted to humans. This may help eliminate the source of infection among the three elements of infectious disease transmission and hence protect humans and animals and prevent the virus from spreading in special places such as pig farms where people and animals are in close contact. This is also in line with ONE Health’s goals and philosophy.

## Data availability statement

The original contributions presented in the study are included in the article/Supplementary material, further inquiries can be directed to the corresponding authors.

## Ethics statement

The animal study was reviewed and approved by Animal experimental committee of Laboratory Animal Center, Changchun Veterinary Research Institute (No. IACUC of AMMS-11-2020-006).

## Author contributions

ZG, CL, TL, and WH: conceptualization. ZG and TL: data curation and formal analysis. NJ, TL, WH, and MT: funding acquisition. NJ and CL: supervision. ZG, TL, SF, LL, YJ, ZX, PH, JC, JH, and PX: methodology. ZG: writing–original draft. ZG, CL, and TL: writing–review and editing. All authors contributed to the article and approved the submitted version.

## Funding

This work was supported by the National Key Research and Development Program of China [No. 2021YFD1801103-6]; the National Natural Science Foundation of China [No. 31972719]; and CAMS Innovation Fund for Medical Sciences (2020-12M-5-001).

## Conflict of interest

The authors declare that the research was conducted in the absence of any commercial or financial relationships that could be construed as a potential conflict of interest.

## Publisher’s note

All claims expressed in this article are solely those of the authors and do not necessarily represent those of their affiliated organizations, or those of the publisher, the editors and the reviewers. Any product that may be evaluated in this article, or claim that may be made by its manufacturer, is not guaranteed or endorsed by the publisher.

## Supplementary material

SUPPLEMENTARY FIGURE S1 NiV-sG expression and characterization. **(A,B)** Construction of a pCDNA-3.1-NiV-sG plasmid and the procedure of NiV-sG expression. **(C)** Protein quality was verified using SDS-PAGE, the protein antigenicity was verified using WB, and the purity was determined by thin-layer scanning.
