# Supplementary figures and images for: Assessment of the immunogenicity and protection of a Nipah virus soluble G vaccine candidate in mice and pigs
Source: Front Microbiol. 2022 Oct 6;13:1031523. doi: 10.3389/fmicb.2022.1031523 (PMC9583134; doi:10.3389/fmicb.2022.1031523)

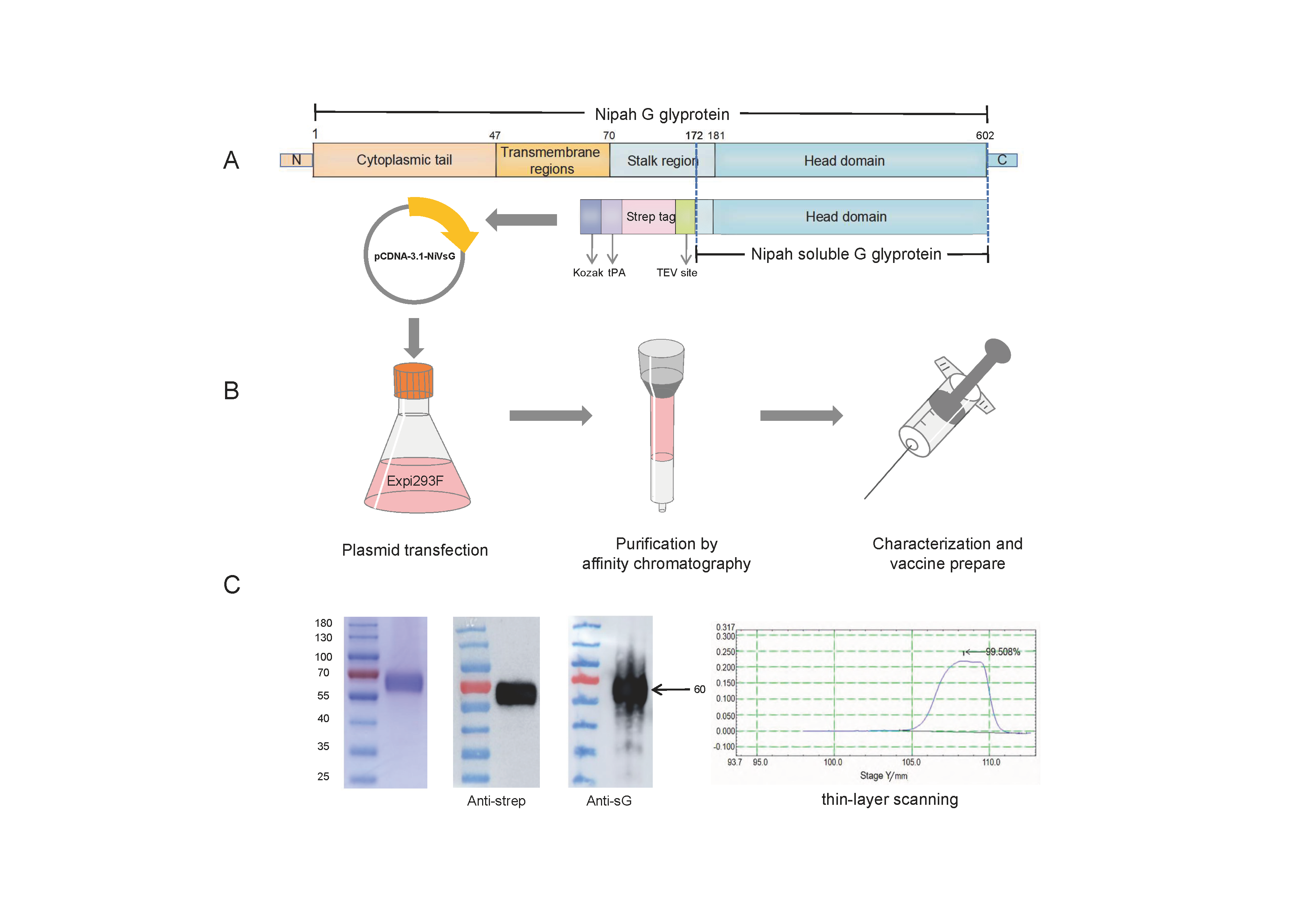

Supplement: SUPPLEMENTARY FIGURE S1 — NiV-sG expression and characterization. (A,B) Construction of a pCDNA-3.1-NiV-sG plasmid and the procedure of NiV-sG expression. (C) Protein quality was verified using SDS-PAGE, the protein antigenicity was verified using WB, and the purity was determined by thin-layer scanning. [file Image_1.TIF]

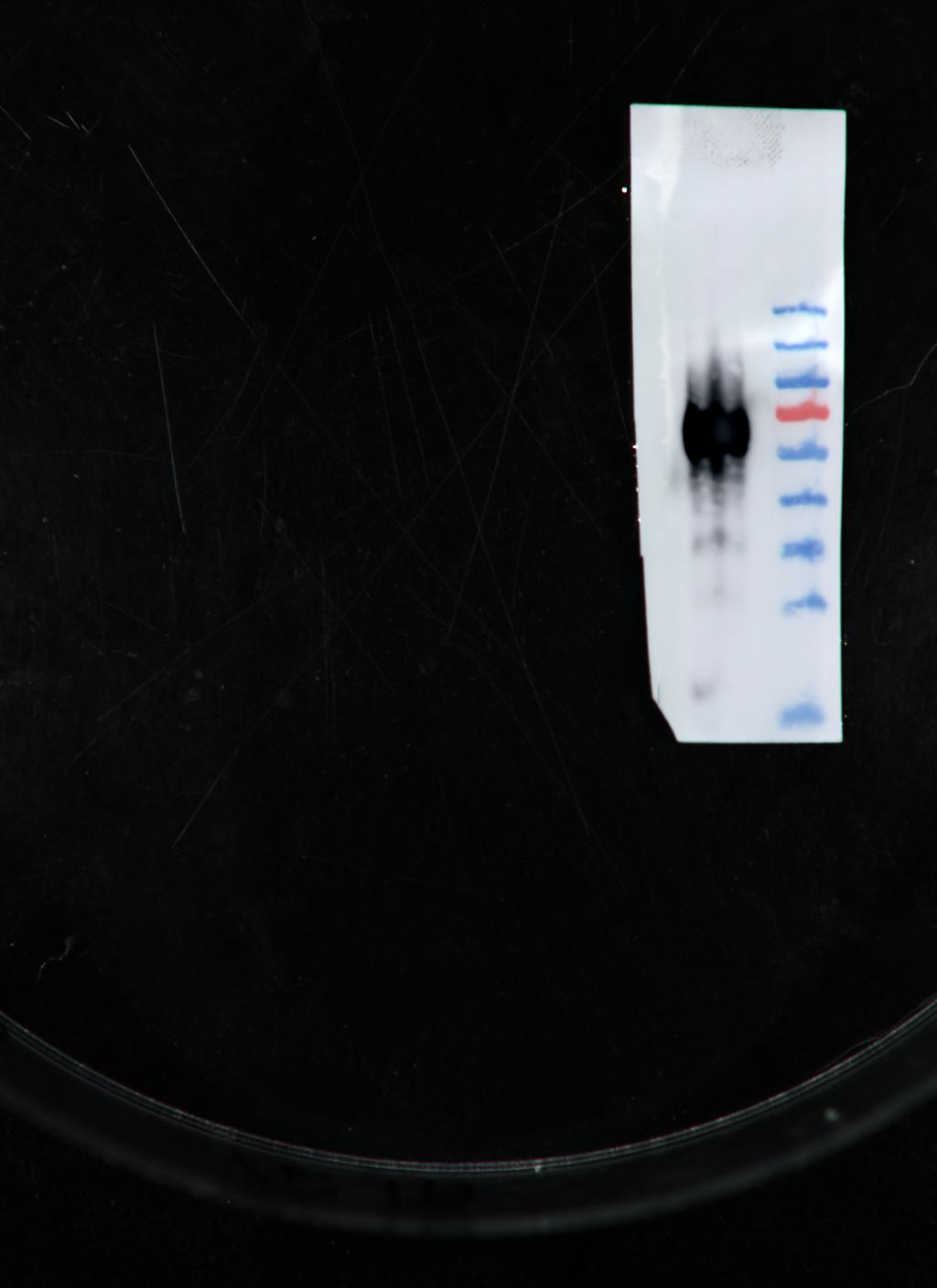

Supplement: Supplementary file 2 [file Image_2.JPEG]

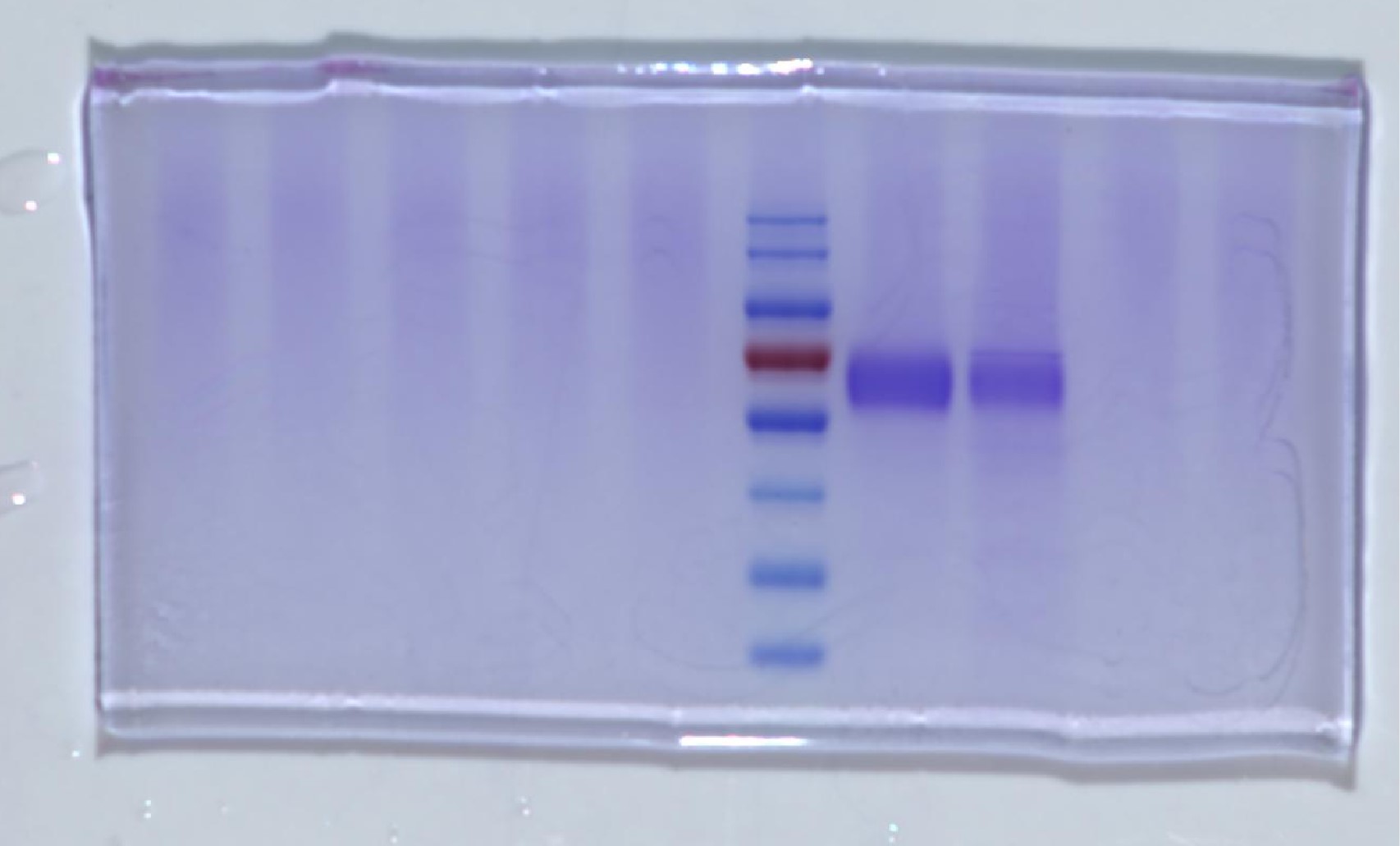

Supplement: Supplementary file 3 [file Image_3.JPEG]

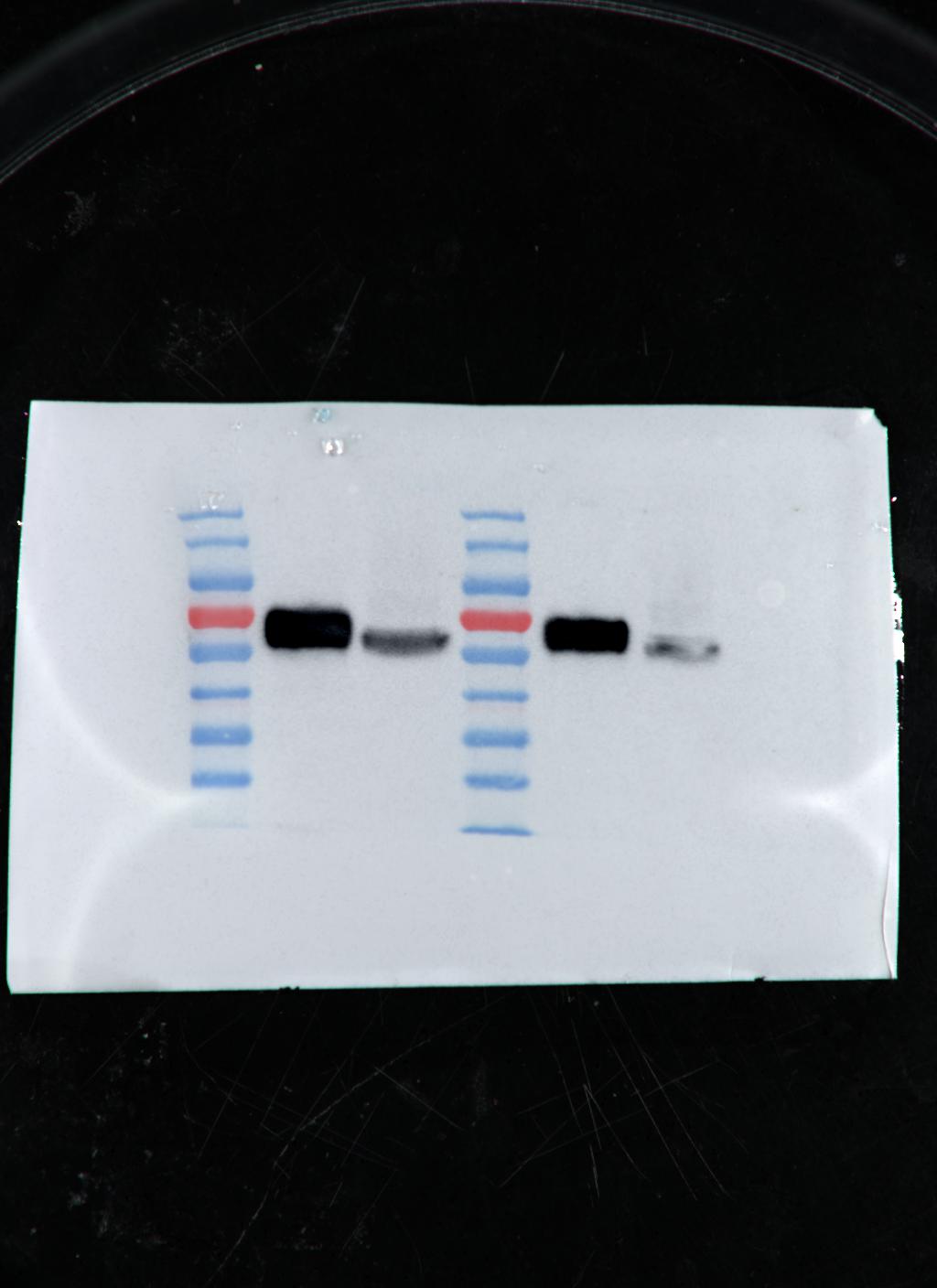

Supplement: Supplementary file 4 [file Image_4.JPEG]
